# Does Altered Cellular Metabolism Underpin the Normal Changes to the Maternal Immune System during Pregnancy?

**DOI:** 10.20900/immunometab20210031

**Published:** 2021-10-04

**Authors:** Benjamin J. Jenkins, April Rees, Nicholas Jones, Catherine A. Thornton

**Affiliations:** Institute of Life Science, Swansea University Medical School, Swansea, Wales SA2 8PP, UK

**Keywords:** immunometabolism, maternal metabolism, pregnancy, Treg, Th2

## Abstract

Pregnancy is characterised by metabolic changes that occur to support the growth and development of the fetus over the course of gestation. These metabolic changes can be classified into two distinct phases: an initial anabolic phase to prepare an adequate store of substrates and energy which are then broken down and used during a catabolic phase to meet the energetic demands of the mother, placenta and fetus. Dynamic readjustment of immune homeostasis is also a feature of pregnancy and is likely linked to the changes in energy substrate utilisation at this time. As cellular metabolism is increasingly recognised as a key determinant of immune cell phenotype and function, we consider how changes in maternal metabolism might contribute to T cell plasticity during pregnancy.

## Introduction

Successful pregnancy involves various maternal adaptations that support the fetus [1]. Metabolic changes allow the preferential passage of nutrients across the materno-fetal interface for optimal fetal growth and development; immune changes ensure tolerance of the fetus [1,2].

**Figure 1:**
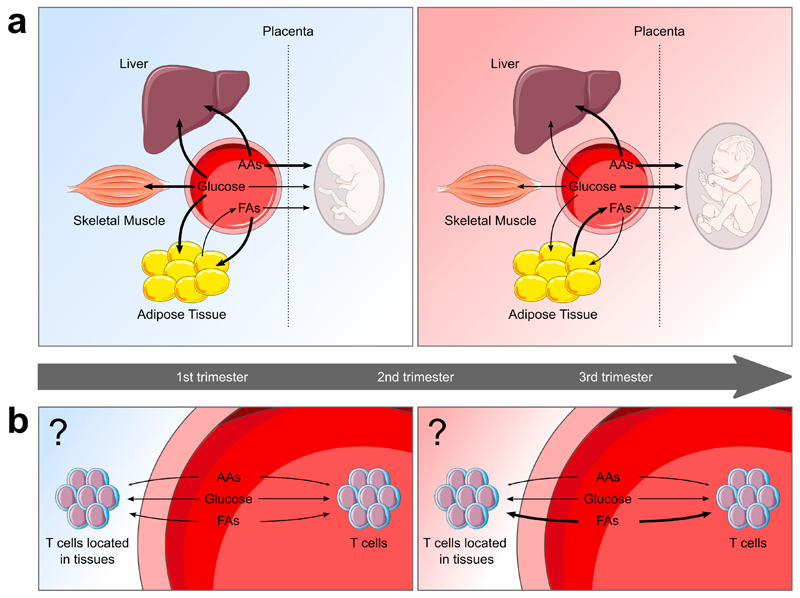
Overview of maternal substrate utilisation across gestation. (**a**) During early pregnancy, there is increased glucose uptake into surrounding tissues. In adipose tissue, glucose is used for the synthesis of triglycerides. Increased breakdown of lipoprotein-triglycerides by lipoprotein lipase results in increased uptake of hydrolytic products into adipose tissue, where they are used in lipogenesis. Lipolytic activity of adipose tissue is also reduced to promote lipid storage. Amino acids also accumulate in tissues, causing hypoaminoacidemia. Transfer of metabolites to the fetus is limited, with amino acids the main contributor. During late pregnancy, glucose uptake into maternal tissues is reduced, and preferentially passes across the placenta to the fetus. In adipose tissue, there is a switch from lipogenesis to lipolysis. Glycerol and free fatty acids (FFA) produced are transferred to lipoproteins, increasing plasma levels of lipoprotein-triglycerides. The fate of amino acid metabolism remains largely unchanged from early pregnancy. (**b**) During early pregnancy there is limited availability of circulating amino acids for uptake by immune cells such as T cells. In the third trimester, the maternal circulation provides T cells with a fatty-acid rich, amino-acid depleted microenvironment. These metabolic conditions could favour Treg and Th2 cell survival and function, as is seen during pregnancy. These changes might not be limited to circulating T cells and could underly the functions of tissue-resident T cells involved in driving pregnancy-related improvements in rheumatoid arthritis and multiple sclerosis. *AA, amino acids; FA, fatty acids; TCA, tricarboxylic acid.*

Maternal metabolic changes during pregnancy are well described in tissues such as adipose, liver and skeletal muscle (summarised in Table 1 and Figure 1). Early pregnancy is characterised by the accretion of metabolic substrates in tissues, which is supported by increases in maternal energy intake [3,4]. In this anabolic phase, insulin promotes the uptake of glucose into tissues where it is either stored [5–7], or in the case of adipose tissue, used for lipogenesis [8,9]. In addition to enhanced lipogenesis, circulating fatty acids (FA) are taken up by and accumulate in maternal adipose tissue [10,11]. Disappearance of amino acids from maternal circulation is also characteristic of pregnancy where nitrogen is either accreted in maternal tissues or the developing fetus [12–14]. However, decreasing insulin sensitivity as pregnancy progresses results in a state of insulin resistance in late pregnancy [15,16]. This promotes a catabolic phase of metabolism wherein glucose preferentially passes to the developing fetus [7] and lipids are hydrolysed to bring about maternal hyperlipidemia [17,18]. Despite gestational changes in glucose utilisation, plasma glucose levels are relatively stable throughout pregnancy [5]. Failure to establish these metabolic changes is associated with numerous gestational complications, such as gestational diabetes mellitus (GDM), pre-eclampsia and cholestasis [19–21].

The influence of cellular metabolism on leukocyte phenotype and function is an increasingly exciting area of immunology. Mechanisms underpinning immune plasticity in pregnancy such as enhanced activity of suppressive or regulatory immune cells and increasing susceptibility of pregnant women to severe responses to some viral infections such as influenza are unknown. Immunometabolism might provide a framework for understanding these dynamic adaptations from pre-conception, through pregnancy and into the postpartum. Although maternal metabolic changes are established in many tissues, it is unknown what effect if any there is on immune cells [26]. This review aims to focus on changes to T cells, especially the regulatory T (Treg) cell and T helper (Th)2 cell biased responses that characterise pregnancy and how changes in maternal energy substrate utilisation might link to changes in the activity of these cells during pregnancy.

## Metabolism and the Immune System

In addition to the metabolic adaptations that are known to occur predominantly in adipose tissue, skeletal muscle and liver, such adaptation in the immune system might underpin the immune plasticity that occurs with pregnancy. Altered energy substrate utilisation is increasingly understood to dictate immune cell fate and function and maternal immunological adaptation might be secondary to the long recognised wider metabolic changes that characterise pregnancy. Expression and activity of pyruvate kinase is reported to be decreased within T cells and neutrophils during normal pregnancy and this will decrease the availability of pyruvate within immune cells [27]. This might suggest that maternal immune cells downregulate their use of glucose for energy substrate production which would alter their function during pregnancy. Indeed, cellular metabolic analysis showed reduced glycolysis in peripheral blood mononuclear cells (PBMCs) of late pregnant women versus non-pregnant women [28]. While this early evidence is suggestive of immunometabolic adaptation in pregnancy, we are a long way from understanding these processes or elaborating their contribution to pregnancy outcome. However, our existing understanding of the immunometabolic characteristics of some immune cell types can provide insight into mechanisms of plasticity during pregnancy with CD4^+^/FoxP3^+^ regulatory T (Treg) cells and Th2 cells perhaps the best examples of this.

## CD4^+^/FoxP3^+^ Regulatory T Cells

Treg cells are important in the control of self-reactive immune cells to maintain immunological self-tolerance [29] and are increased in both the decidua and maternal peripheral blood with pregnancy [30,31]. The majority of the maternal Treg population consists of effector Treg cells not resulting from clonal expansion [32,33]. Reduced numbers of peripheral Treg cells have been associated with several adverse pregnancy outcomes including recurrent miscarriage and preeclampsia [34,35]. Mouse models of pregnancy have demonstrated that Treg cells are required to mediate maternal tolerance to the fetus [36]. Their absence is characterised by significant lymphocyte infiltration and haemorrhage at the materno-fetal interface, which ultimately results in abnormal fetal development and gestational failure [36]. Additionally, aberrant Treg function also results in abortion and the transfer of Treg cells from healthy pregnancies to these abortion-prone mice improves pregnancy outcome [37]. This highlights the important role for Treg cell biased responses in ensuring successful pregnancy.

Maternal Treg cells are likely to play an important role in facilitating peripheral immune tolerance as evidenced by improved outcome of some autoimmune diseases during pregnancy. For example, decreased disease severity and complete remission have been seen in pregnant women with rheumatoid arthritis (RA) [38,39]. RA disease severity is inversely correlated with maternal Treg cell numbers in humans and Treg cells from pregnant mice alleviated disease when transferred into non-pregnant RA mice [40,41]. Pregnancy-induced amelioration of multiple sclerosis is associated with the expansion and enhanced function of maternal Treg cells [42–44]. In contrast maternal Treg cells can confer increased susceptibility to infection through their immunosuppressive function [45].

**Figure 2:**
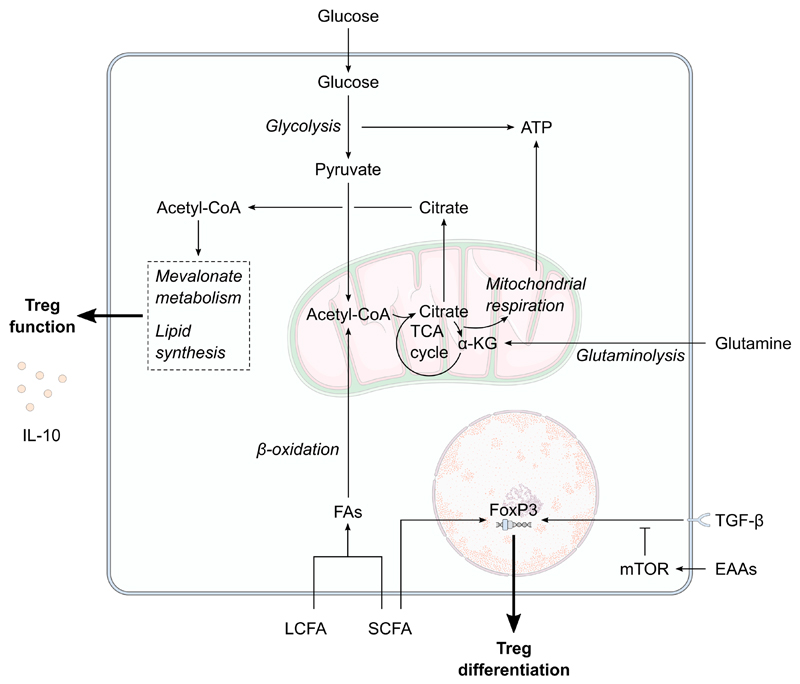
The potential effect of maternal metabolism on Treg differentiation and function. Decreased extracellular amino acids concentrations combined with increased transforming growth factor-β concentrations during pregnancy would enhance the FoxP3-mediated regulatory T cell (Treg) differentiation. Short-chain fatty acids amongst the increased maternal extracellular fatty acid pool would also enhance Treg differentiation. Increased circulating fatty acid concentrations could increase the availability of cellular acetyl-coA. Flux of acetyl-coA through mevalonate metabolism and lipid synthesis is vital for Treg function, such as interleukin-10 (IL-10) production, and Treg survival. α-KG, α-ketoglutarate; *EAAs, essential amino acids; FA, fatty acid; IL-10, interleukin-10; LCFA, long-chain fatty acid, mTOR, mechanistic target of rapamycin; SCFA, short-chain fatty acid; TCA, tricarboxylic acid; TGF-β, transforming growth factor-β; Treg, regulatory T cell.*

Together, this provides evidence that changes to maternal Treg cell function are not limited to the fetal-placental interface but also occur systemically during pregnancy and are best exemplified by measures in maternal peripheral blood although in humans these observations relate predominantly to relative changes in abundance rather than functional readouts. However, it remains unknown what the underlying mechanisms are that drive increased Treg cell function during pregnancy. Given our understanding of the role of metabolism in Treg cell development and function more generally, it is likely that the altered metabolism that occurs in normal pregnancy as described above contributes to changes in Treg cell function (Figure 2). Metabolism likely interacts with hormone-dependent and fetal antigen-driven changes to Treg responses in pregnancy; indeed placental hormones are key drivers of the metabolic adaptations summarised above and in Table 1. Increased Treg numbers are common in both allogeneic and syngeneic pregnancies highlighting the role of pregnancy hormones [36], with a greater increase in allogeneic pregnancies [46], suggesting that fetal alloantigens make a telling contribution. While the impact that the local metabolic environment might have on Treg fate and function has yet to be elucidated in pregnancy, the following subsections will highlight areas of the literature that suggest it could contribute to gestational changes in Treg responses.

### Amino Acids and Treg Differentiation

There are numerous features of the maternal metabolic environment that might favour enhanced Treg function. Maternal hypoaminoacidemia presents early in pregnancy and persists throughout gestation [12,23,24]. Amino acid deprivation can influence Treg differentiation and has been highlighted by the induction of a Treg phenotype during tryptophan starvation [47]. Tryptophan depletion and the immunoregulatory tryptophan catabolite kynurenine modulate FoxP3 expression through general control nonderepressible (GCN)-2 kinase activation and TGF-β signalling to induce a regulatory phenotype [47]. Plasma tryptophan levels are decreased during the 2nd and 3rd trimesters, decreasing as pregnancy progresses [48], which might suggest that the maternal circulation provides a favourable microenvironment for differentiation towards Treg cells. Although kynurenine levels in maternal plasma remain stable during pregnancy, the increase in kynurenine to tryptophan ratio with each trimester [48] could further promote the induction of CD4^+^/FoxP3^+^ T cells. Notably, this scenario very much reflects our focus on the changes that occur peripherally in pregnant women especially as pregnancy progresses. Local metabolic changes within the early pregnant uterus that might underpin pregnancy-favouring behaviours of Treg cells and other key effectors of implantation and placentation success such as decidual NK cells remain to be established. Similarly, perturbations in these that might then link to adverse pregnancy outcomes such as miscarriage remain to be elucidated.

The availability of other essential amino acids can also influence FoxP3^+^ Treg cell differentiation from CD4^+^ T cells and the concentration of TGF-β required for differentiation is lower in conditions of essential amino acid deprivation [49]. Of the essential and conditionally-essential amino acids that drive FoxP3 induction at limited concentrations in synergy with TGF-β, four have reduced plasma concentrations during pregnancy—valine, arginine, phenylalanine and tryptophan [50]. Under conditions of amino acid deprivation, mechanistic target of rapamycin (mTOR) regulates differentiation of CD4^+^ T cells to FoxP3^+^ induced Treg (iTreg) cells [49,51]. Inhibition of mTOR favours Treg cell differentiation, as mTOR activity limits FoxP3 induction by key transcription factors SMAD and FOXO. TGF-β-induced SMAD3 and SMAD4 function and nuclear localisation of FOXO1 and FOXO3 are inhibited by mTORC1 and mTORC2 respectively [52]. The activation of both mTORC1 and mTORC-2 is controlled directly by extracellular amino acids [53,54]. Indeed, essential amino acid deprivation reduces S6 phosphorylation in CD4^+^ T cells which then increases Treg cell differentiation [49]. Increased TGF-β concentrations in maternal serum during pregnancy [55] could also play a significant contribution towards Treg induction, especially when coupled with hypoaminoacidemia.

Additionally, reduced levels of glutamine can also promote Treg differentiation from CD4^+^ T cells through inhibition of mTOR [56,57]. The effects of glutamine deprivation on mTOR activity can be rescued by the addition of α-ketoglutarate (α-KG), the product of glutaminolysis, therefore it is possible that this pathway underpins the modulation of mTOR by glutamine [56]. Given that the same restorative effect of α-KG was not observed in human iTreg cells [57], glutamine deprivation might also influence differentiation via alternative mechanisms. α-KG can further regulate Treg differentiation from CD4^+^ T cells through epigenetic modification of FoxP3 [58]. Hypermethylation within the Treg-specific demethylated region of the FoxP3 gene by 2-hydroxyglutarate, derived from α-KG, inhibits the expression of the FoxP3^+^ Treg phenotype [58]. Therefore, glutamine-deprived conditions would limit the availability of α-KG for conversion to 2-hydroxyglutarate and would maintain the demethylated status of the FoxP3 Treg-specific region, allowing its transcription and the consequent differentiation to Treg cells. Glutamine could also affect Treg differentiation from CD4^+^ T cells independent of glutaminolysis as inhibition of glutaminase, responsible for the conversion of glutamine to glutamate, does not induce the differentiation of Treg cells [59]. Plasma glutamine levels are reduced during the third trimester, whilst there is also a trimester-by-trimester decrease in the availability of branched chain amino acids (BCAA) that can act as a nitrogen pool for glutamine synthesis [50,60]. Glutamine uptake in CD4^+^ T cells is also required for leucine uptake via SLC7A5-CD98 amino acid transporter [61]. Leucine import results in activation of mTORC1, favouring differentiation towards other non-Treg subsets [61]. Decreased extracellular glutamine availability, as seen in pregnancy, could therefore limit leucine-mediated mTOR activity to skew towards a regulatory phenotype. Trimestral decreases in plasma leucine [50] could further hamper T cell mTOR activation and favour induction of Tregs. Thus, the amino acid-depleted environment of the maternal circulation during pregnancy potentially favours Treg cell differentiation.

### Fatty Acids and Treg Differentiation

Fatty acid (FA) metabolism also plays an important role in controlling the fate of Treg cells and the enhanced efflux of lipid breakdown products from maternal adipose tissue during the third trimester could support Treg cell expansion. Exogenous FA uptake by iTreg cells allows sustained proliferation and favours a regulatory phenotype [62]. iTreg cells are less dependent on acetyl-CoA carboxylase (ACC)1 for their development and can instead convert exogenous FAs into palmitate for phospholipid synthesis [62]. ACC1 inhibition and its effect on de novo FA synthesis does not affect iTreg development and promotes differentiation towards a regulatory lineage [62]. In particular, the oxidation of FAs taken up by CD4^+^ T cells is important for their development as iTreg cells—addition of FAs alone enhances differentiation towards the regulatory lineage whilst addition together with TGF-β further skews differentiation [63]. Underlying this process are the actions of both mTOR and AMP-activated protein kinase (AMPK)—reduced mTOR activity and heightened AMPK activity enhancing lipid metabolism which in turn increases the generation of CD4^+^/FoxP3^+^ T cells [63]. Consequently, pharmacological activators of AMPK such as metformin and 5-aminoimidazole-4-carboxamide ribonucleotide (AICAR) are capable of enhancing Treg differentiation [63,64]. Inhibiting carnitine palmitoyltransferase 1a (CPT1a), the rate controlling enzyme of long-chain FA oxidation, with etomoxir blocks Treg differentiation from CD4^+^ T cells and outlines the importance of lipid oxidation [63]. Indeed, direct activation of AMPK by AICAR promotes Treg differentiation from CD4^+^ T cells through increased mitochondrial biogenesis and FA uptake and depends on the latter to exert its effect on differentiation [64]. However, emerging understanding of the off-target effects of etomoxir need to be taken into account when interpreting these data [65]. The metabolic sensor LKB1, upstream of AMPK, has also been identified as an important regulator of T cell metabolism and survival [66,67]. Compromised OXPHOS and ATP production upon *LKB1* deletion ultimately resulted in reduced Treg numbers [66,67]. However, it is understood that LKB1 function in Treg cells occurs independently of conventional AMPK pathways and instead mediates MAPK signalling [66].

Short-chain FAs (SCFAs) also promote Treg differentiation [68]. SCFAs polarise CD4^+^ T cells towards a regulatory phenotype through inhibition of the JNK1 and the p38 pathway [68]. Increasing levels of the SCFA β-hydroxybutyrate have been reported across pregnancy [50]. The sodium salt of butyrate can regulate the Th17/Treg balance to favour CD4^+^/FoxP3^+^ Treg cells [69]. Nuclear translocation and activation of the Nrf-2/HO-1 pathway is critical in sodium butyrate-mediated modulation of Th17 differentiation and the reciprocal increase of CD4^+^/FoxP3^+^ T cells [69]. Additionally, butyrate can act directly on peripheral and splenic naïve CD4^+^ T cells to enhance their differentiation to FoxP3^+^ Treg cells by stabilising the acetylation of the critical transcription factor FoxP3 [70,71]. The SCFA propionate can act on CD4^+^ T cells in the same manner as butyrate and presents an alternative method through which SCFAs can regulate Treg differentiation [70]. As maternal plasma is enriched with SCFAs [50], it is plausible that this could contribute to the expanded Treg population observed during pregnancy. The contribution of the maternal gut microbiome to these SCFAs is beyond the scope of this review but there is immense interest in the relationship between the maternal microbiota and immune programming of the offspring [72]. Together, our current understanding of both maternal metabolism during pregnancy and the role of metabolism underlying Treg differentiation would suggest that the maternal circulation provides a highly favourable environment for Treg cells—especially during the third trimester. Given that this population of T cells is increased during pregnancy, it is possible that this occurs due to the amino acid-deprived landscape of maternal peripheral blood, and is further supplemented by the increased appearance of FAs during the third trimester.

### Hormonal Regulation of Treg Function

Further to the aforementioned effect that pregnancy hormones have on Treg cells, metabolic hormones such as leptin can also influence Treg responses. Treg cells express higher amounts of the leptin receptor (ObR) than other T cell subsets, which suggests that leptin might have a greater influence in shaping their functional output [73]. Indeed, leptin negatively regulates Treg proliferation, but does not have a significant effect on the suppressive capacity of these cells [73]. The absence of leptin, or chronic blockade of its receptor, increases Treg cell numbers which has led to resistance in models of autoimmune diseases [73,74]. Leptin also plays some important roles during pregnancy—recombinant leptin improved conception in infertile mice, whilst regulatory roles in immunomodulation, angiogenesis and nutrient transport have been described in the placenta [75,76]. Usually produced in adipose tissue, placenta-derived leptin emerges as a novel pregnancy hormone and during the second and third trimester plasma leptin levels become elevated including a contribution of the placenta as a pregnancy-specific source of leptin [77,78]. Leptin resistance develops during the second trimester and has a role in supporting the growth of the fetus [79,80], whilst maternal obesity has been associated with further leptin resistance in the placenta [81]. While the leptin-rich environment that presents during the second and third trimester might be expected to contradict the known effects of leptin on Treg number, the accompanying leptin resistance would favour increased Tregs [73,74], although we do not know if this leptin resistance extends to the immune system and more specifically Treg cells.

### Fatty Acids and Treg Function

Metabolism not only dictates Treg differentiation but also controls Treg cell effector function. Thymic Treg (tTreg) cells engage glycolysis upon activation through TNF receptor 2 co-stimulation, which is required for their expression of key transcription factors such as FoxP3, as well as their suppressive function [82]. As maternal plasma glucose levels are limited during pregnancy [5], our focus has shifted to other elements of metabolism, such as fatty acids and amino acids, whose changes are more profound during pregnancy and are key contributors to cellular metabolic pathways. Treg cells with significantly reduced lipid synthesis have reduced survival and attenuated immunosuppressive function [66,67,83]. Upon stimulation through PD-1 and CTLA-4 and induction of FoxP3 expression, iTreg cells prepare for effector function by lowering the rate of glycolysis whilst concomitantly increasing oxidative metabolism and FA oxidation [84]. iTreg cells fail to upregulate Myc upon stimulation which results in restrained glycolysis [85]. Inhibition of either OXPHOS or FA oxidation diminishes the suppressive function of iTreg cells and thus underlines the importance of metabolism in directing a Treg response [85,86]. In a mixed population of iTreg and tTreg cells, uptake and oxidation of exogenous FA contributed to OXPHOS and favoured Treg stability [87]. Inhibition of the mevalonate pathway through deletion of HMG-CoA reductase also causes defective Treg cell function in both iTreg and tTreg cells and culminates in an autoimmune disease phenotype [83,88,89]. LKB-1 deficient iTreg cells produce less IL-10 and have decreased surface expression of effector molecules such as CTLA-4, PD-1, CD39 and CD73, instead enhancing their production of inflammatory cytokines which ultimately results in failure to inhibit autoimmunity [83]. The mevalonate pathway is impaired in LKB-1 deficient iTreg cells and inhibition of HMG-CoA reductase induces a similar inflammatory phenotype to that of LKB1 deletion [83]. Suppressive iTreg function is restored upon treatment with mevalonate and geranylgeranyl pyrophosphate (GGPP) [83]. Increased plasma lipid concentrations, as seen during late pregnancy, would enhance substrate availability to these pathways and might explain enhanced gestational Treg cell function as pregnancy progresses.

Recently, fatty acids have emerged as key regulators of Treg cell mitochondrial health [90,91]. Expression of CD36, a fatty acid translocase, is required for the immunosuppressive function and survival of intratumoural Treg cells [90]. CD36 also modulates the mitochondrial fitness of both intratumoural Treg cells and iTreg cells and supports their metabolic adaptation within the microenvironment [90]. Lipid chaperones responsible for the uptake and intracellular transport of lipids, such as fatty acid binding protein 5 (FABP5) are also implicated in maintaining mitochondrial health in iTreg cells [91]. Inhibition results in impaired oxidative respiration, loss of cristae structure and ablated lipid metabolism [91]. Under these circumstances there was a reduction in iTreg cell number, however, suppressive function was augmented through the release of mitochondrial DNA caused by mitochondrial dysfunction [91]. The resulting cGAS-STING signalling and subsequent type I IFN signalling promote IL-10 expression and drive suppressive Treg function [91].

### Amino Acids and Treg Function

Amino acids play a more reserved role in orchestrating suppressive Treg function. Glutamine depletion produces iTreg cells with suppressive function such as IL-4 production, however, these cells also express pro-inflammatory cytokines IL-17 and IFNγ [57]. This might suggest that balanced amino acid levels are required for successful regulatory function. Collectively, these data support the hypothesis that maternal immunological adaptation is secondary to changes in metabolism and energy substrate utilisation. It is important to note that the majority of the studies discussed in this manuscript analyse the fate and function of iTreg cells, with those concerning tTreg cells highlighted. Whether the concepts addressed throughout this section apply to both iTreg and tTreg cells is not known, but would be an important factor to consider in any future research. It is also important to acknowledge that other Treg populations, such as those localised at the decidua, might be differentially affected compared to the peripheral blood Treg cells discussed. For example, little is known about that the metabolic environment at the materno-fetal interface, therefore understanding the effect that this local metabolic environment might impose on Treg cells could be fundamental to elucidating reduced Treg numbers in women with recurrent pregnancy loss and other adverse pregnancy outcomes. As yet there are no experimental data to support this hypothesis other than the coalignment of what we know about changing Treg cell number in pregnancy and the changes to substrate availability. Detailed investigation of the metabolic changes to Treg cells that accompany pregnancy normally and in adverse pregnancy outcomes such as miscarriage and preeclampsia are certainly warranted.

## Th2 Cells

In addition to Treg cells, there is an increase in the activity of Th2 cells during pregnancy. T helper (Th) cells have been long characterised by their cytokine profile and this allows their classification into different subsets [92]. Th2 responses are characterised by the release of IL-4, IL-5, IL-9 and IL-3. Naïve CD4^+^ T cells differentiate to a Th2 phenotype through activation of signal transducer and activator of transcription (STAT)6 and GATA3. Increased Th2 cell activity occurs at both the fetal-placental interface and in peripheral blood and is measured as a ratio of the activity of Th2 cells compared to Th1 cells. During pregnancy, there is an increase in the secretion of type 2 immune cytokines—so origins possibly other than T cells such as IL-4, with a relative increase compared to cytokines such as IFNγ and IL-2 [93,94]. This likely occurs due to changes at the protein level that reflect increased IL-4 mRNA expression in T cells whilst IFNγ mRNA expression is decreased [95]. This ratio remains throughout gestation and reverts to non-pregnant levels postpartum [96,97]. Some studies suggest that Th2 activity declines as early as the latter stages of labour [98]. Although enhanced Th2 activity has been demonstrated throughout human pregnancy, the complete absence of a Th2 response does not affect allogenic mouse pregnancy, which suggests that Th2 cells are not essential in maintaining the materno-fetal tolerance—at least in mice [99].

### Amino Acids and Th2 Responses

Global changes in maternal metabolism also could underpin the Th2 biased responses that are observed during pregnancy as metabolic pathways are key regulators of both Th2 differentiation and function. Characteristic reductions in plasma amino acid concentrations during pregnancy, particularly reduced glutamine [12,50], potentially tips the Th1/Th2 ratio in favour of Th2 cells. Treatment of PBMCs with high concentrations of glutamine in vitro impairs Th2-like responses through suppression of IL-10 production and augmenting IFNγ production [100]. These changes are realised physiologically as dietary glutamine shapes the Th1/Th2 balance towards Th1 cells over Th2 cells, reflected by increased IL-2 production and decreased IL-4 production [101]. In fact, Th2 cells are not dependent on glutamine and rather have improved function in its absence [61]. Deletion of ASCT2 enhanced the generation of Th2 cells, increasing the expression of both GATA3 and IL-4 [61]. A possible mechanism of action for glutamine-mediated T cell regulation is its inhibition of cytosolic phospholipase A2 activity [102]. Th2 cytokine production is reduced in the presence of glutamine which culminates in failure to recruit neutrophils and eosinophils to inflamed airways [102]. Cytosolic phospholipase A2 is important in facilitating the release of arachidonic acid from glycerophospholipids for their incorporation into Th2-related inflammatory mediators.

The contribution of tryptophan metabolism to Th2 function has already been demonstrated in the setting of murine pregnancy [103]. Decreased tryptophan metabolism in the uterus balances the Th1/Th2 ratio in the favour of Th1 cells which increases the likelihood of pregnancy failure [103]. In human pregnancy, low levels of tryptophan but maintained levels of its metabolites such as kynurenine suggest increased tryptophan metabolism and utilisation of the metabolites [48] and would present a Th2 favouring milieu. The absence of tryptophan catabolising enzymes in the lung impaired Th2-mediated inflammatory airway responses with reductions in IL-5, IL-13 and IL-4 production [104]. The activity of the tryptophan-catabolising enzyme indoleamine-2,3-dioxygenase (IDO) is thought to be increased during pregnancy, playing an important role in immune activation [105]. Additionally, tryptophan catabolism deficiency had less of an impact on Th1 responses [104]. Interestingly, metabolites of the kynurenine pathway cause apoptosis of Th1 but do not impact Th2 survival [47]. Together, these data suggest that increased flux through tryptophan catabolism pathways supports Th2 responses in the Th1/Th2 balance. Thus, the amino acid landscape of pregnancy could support the Th2-skewed Th1/Th2 ratio that is observed.

### Fatty Acids and Th2 Responses

Lipid metabolism is also implicated in Th2 cell expansion and function. Inhibition of ACC1 impairs Th2 induction and underlines the importance of lipid synthesis in mediating Th2 responses [62,106]. Lipid pathways also play an important role in Th2 development. Upon stimulation and subsequent activation of CD4^+^ T cells, FA uptake is increased and is essential for successful proliferation [106]. Lipid synthesis through ACC1 was also important for proliferation and made a significant contribution to glycolysis [106].

Peroxisome proliferator-activated receptor (PPAR)γ—the master regulator of lipid metabolism and storage—entwines cellular metabolism with Th2 effector function. PPARγ inhibition inflicted the same metabolic and functional effects on CD4^+^ T cells as ACC1 inhibition, suggesting that PPARγ-mediated metabolic reprogramming directs the activation of Th2 cells [106]. Gene expression analysis has revealed that activated Treg cells are enriched in genes associated with lipid metabolism [107]. There are more PPARγ binding sites on open chromatin at loci that facilitate enhanced expression of the lipid-associated target genes needed for glucose metabolism and promoting Th2 functions such as recruitment of eosinophils to inflamed airways [107]. Included in the target genes for PPARγ binding are IL-5 and IL-13, and key Th2 transcription factor GATA3 [108]. Th2 cells lacking PPARγ failed to mount a Th2 response to allergic challenge in the airways of mice [109]. PPARγ deletion reduced the production of IL-5 and IL-13 by Th2 cells and reduced the presence of eosinophils and mucus-secreting goblet cells, culminating in an impaired pathogenic response [109]. Similar responses were also seen in other mouse models of allergic airway inflammation and *H. polygyrus* infection [109,110], emphasising the importance of PPARγ in protective immunity. IL-33 plays a critical role in PPARγ signalling in Th2 cells. IL-33R ligation upregulates the expression of PPARγ, which itself can upregulate the expression of IL-33R subunit ST2, creating a positive feedback loop [109,110].

The role of PPARγ activity in IL-4 production remains unclear, with reports that it can enhance [110], suppress [111] or have no effect [109] on IL-4 expression. It has also been proposed that PPARγ can influence Th2 response indirectly via the actions of dendritic cells [110]. Expression of PPARγ by dendritic cells is controlled by IL-4 and IL-33 and drives polarisation of T cells towards a Th2 phenotype [110]. Production of IL-9 also has been associated with PPARγ, giving rise to a subset of T cells that also produce increased amounts of IL-5 and IL-13 [112]. However, activation by TGF-β and IL-4, which enhance the expression of PPARγ, promotes a Th9 phenotype where Th2 cytokines are downregulated [112]. The ability of PPARγ to influence metabolic pathways likely underpins its role in controlling Th2 functions. In the absence of PPARγ, FA uptake was attenuated with mediators such as FABP5 downregulated [106], whilst carbohydrate synthesis, metabolite transport, lipid storage and lipolysis were also all affected [109].

**Figure 3:**
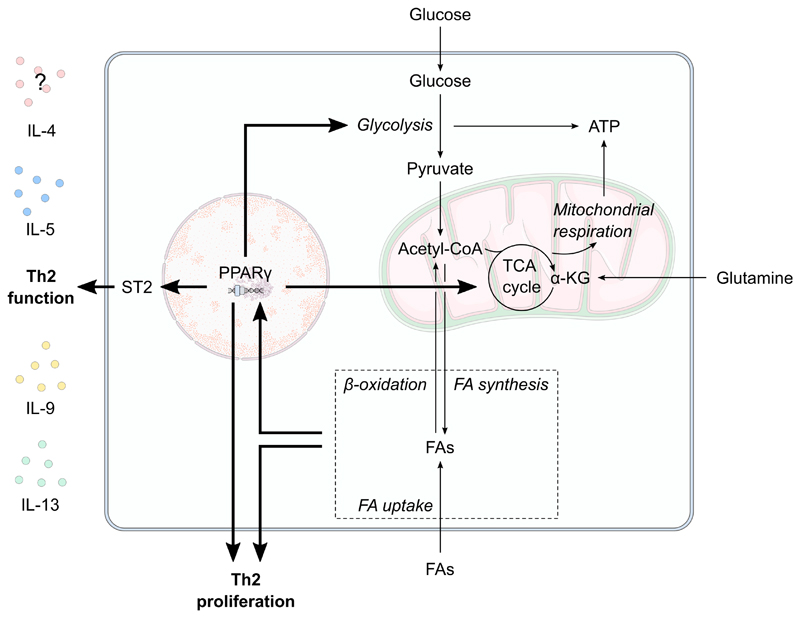
The potential effect of maternal metabolism on Th2 proliferation and function. Peroxisome proliferator-activated receptor (PPAR)-γ is central to T helper 2 (Th2) cell glycolytic and oxidative metabolism, function and proliferation. Pregnancy is associated with increased circulating fatty acid (FA) levels. Enhanced lipid metabolism, such as increased fatty acid uptake, oxidation and synthesis, likely influences PPARγ activity to increase Th2 proliferation. PPARγ also promotes ST2 expression to increase the expression of Th2-related cytokines such as IL-9, IL-13, IL-15 and possibly IL-4. *α-KG, α-ketoglutarate; FA, fatty acid; IL, interleukin; TCA, tricarboxylic acid; TH2, T helper 2 cell.*

The influence of SCFAs on Th2 function remains unclear. Early work suggests that SCFAs have a negative effect on Th2 responses, with long-term defects on IL-5 and IL-13 production upon propionate treatment [113]. However, recently various C2–4 SCFAs have been shown to increase the expression of IL-5 following FFAR3-mediated signalling [114]. In vivo, SCFA treatment materialises as enhanced Th2 cytokine production and a consequent increase in eosinophil recruitment [114]. The hyperlipidemic maternal phenotype appears tailored for enhanced Th2 cell function (Figure 3). Increased lipid availability would provide the substrates necessary for enhanced type 2 cytokine production, as is seen in normal pregnancy, and perhaps explains the mechanisms underpinning the Th2 biased responses seen during pregnancy. However, as for Tregs above there are no direct data to support this postulated, but such studies warrant investigation.

## Concluding Remarks

Metabolic adaptation during pregnancy is essential for successful fetal growth and development. This is achieved through many tightly regulated processes occurring as a result of the overarching increase in insulin resistance as gestation progresses. The effect of these processes on tissues such as the liver, adipose and skeletal muscle are well described, however it is unknown how these processes might affect the immune system. Immune plasticity is required during pregnancy, both to ensure tolerance of the semi-allogenic fetus and to protect mother and fetus from infection. Metabolism has emerged as a central regulator of immune cell phenotype and function, therefore it is increasingly likely that maternal immunological adaptation is governed by wider metabolic changes characteristic of gestation. Understanding the pathways underlying changes in immunometabolism and immune cell fate and function could reveal potential therapeutic targets to alleviate disease severity during pregnancy, benefitting the long-term health of both the mother and newborn. This will also reveal the spectrum of normal immunometabolic programming and plasticity in humans that could be harnessed for therapeutic benefit in disease settings outside of pregnancy.

## Figures and Tables

**Table 1:** Overview of known metabolic changes that occur during pregnancy.

Metabolite	Change during pregnancy	Species	Reference
Glucose	Plasma glucose concentrations remain at pre-gravid levels throughout pregnancy	Human	[5]
	Glucose uptake into maternal tissues decreases in the 3rd trimester	Human	[7]
	Glucose oxidation is reduced during the 3^rd^ trimester	Human	[7]
	Increased utilisation of glucose for lipid synthesis, which declines rapidly towards the end of pregnancy	Rat	[22]
Fatty acids (FA)	Increased accumulation of FAs in adipose tissue in the first and second trimester	Human	[11]
	Elevated lipogenesis in maternal tissues during the first and second trimester	Rat	[8]
	Increased lipoprotein lipase (LPL) activity during the first and second trimester drives FA uptake by surrounding tissues	Human	[10]
	Decreased LPL and hepatic lipase activity during the third trimester reduces lipid uptake into tissues	Human	[10]
	Increased lipolysis occurs within adipose tissue at the end of pregnancy and results in hyperlipidemia	Rat	[18]
	Circulating lipoprotein levels increase throughout pregnancy	Human	[10]
Amino acids	Hypoaminoacidemia occurs early in pregnancy and remains throughout	Human	[12,23,24]
	Relative urea production and excretion is reduced throughout pregnancy	Human	[13]
	Protein oxidation is reduced at the end of pregnancy	Human	[25]
